# Engineered probiotics platform for oral delivery of antibody as a high-compliance alternative for immune-mediated inflammatory diseases

**DOI:** 10.1016/j.xcrm.2025.102523

**Published:** 2025-12-19

**Authors:** Jia Liu, Lexuan Wang, Bingyu Pang, Yichenxi Shi, Yang Chen, Ruili Zhang, Shuai Shao, Chaoqiang Qiao, Zhongliang Wang

**Affiliations:** 1 Engineering Research Center of Molecular & Neuroimaging, Ministry of Education, School of Life Science and Technology, Xidian University, Xi’an 710126, P.R. China; 2 Department of Dermatology, Xijing Hospital, Fourth Military Medical University, Xi’an, Shaanxi 710032, China; 3 Guangzhou Institute of Technology, Xidian University, Guangzhou 510555, P.R. China

**Keywords:** engineered probiotics, oral antibody delivery, immune-mediated inflammatory diseases, psoriasis

## Abstract

Antibody-based therapies have transformed the management of immune-mediated inflammatory diseases (IMIDs), but the need for frequent injections often leads to inadequate patient adherence and suboptimal long-term disease control. To address this challenge, we develop AIDEN (aid for IMIDs: engineered EcN), an engineered probiotic platform that enables oral delivery of therapeutic antibodies using synthetic biology. In this study, we assess the efficacy of AIDEN-IL17, a variant designed to secrete single-chain variable fragments targeting interleukin-17A (IL-17A), in murine models of psoriasis and inflammatory bowel disease. AIDEN-IL17 exhibits stable gut colonization and sustained *in situ* antibody production, resulting in moderate reduction of systemic IL-17A levels and significant amelioration of disease symptoms. Notably, the AIDEN platform is modular and adaptable for delivering a broad range of antibody therapeutics, offering a promising, patient-friendly strategy for the treatment of IMIDs.

## Highlights

• Construct AIDEN-IL17 for *in situ* on-demand production of anti-IL-17A scFvs
• AIDEN-IL17 secretes functional scFvs for prolonged IL-17A neutralization
• AIDEN-IL17 offers a cost-effective and convenient alternative for IMIDs

## Graphical abstract

**Figure:**
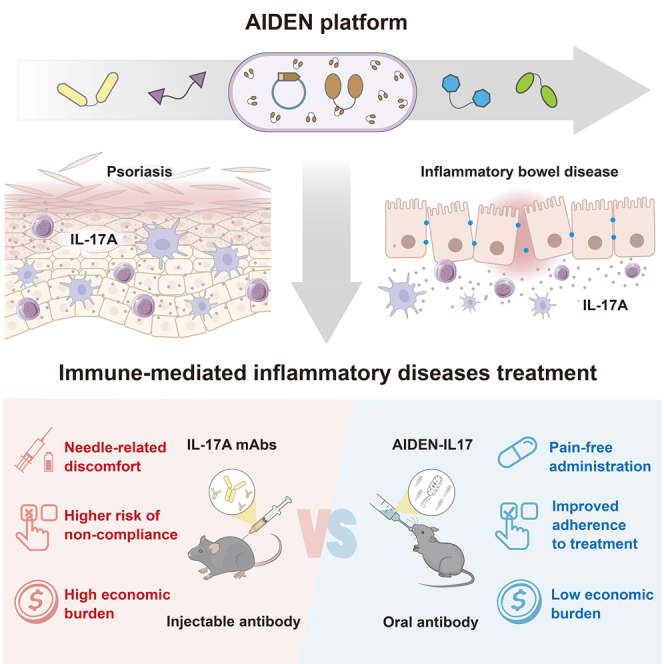

## Teaser

Liu et al. develop an engineered probiotics platform (AIDEN) to overcome the challenge of inadequate patient compliance for lifelong management of immune-mediated inflammatory diseases. AIDEN-IL17, a variant targeting IL-17A, exhibits moderate yet sustained neutralization of IL-17A in murine models and controls inflammation progression in psoriasis and colitis.

## Introduction

Immune-mediated inflammatory diseases (IMIDs), characterized by shared pathogenetic features like “public” immune pathways, are frequently incurable and necessitate lifelong management.^1^^,^^2^ Antibody-based therapies targeting key mediators, such as tumor necrosis factor alpha (TNF-α), interleukin-17 (IL-17), IL-23, or their receptors, have significantly advanced the treatment of IMIDs owing to their specificity, neutralization capacity, and extended half-life.^3^^,^^4^ Additionally, strict adherence to prescribed medication regimens is critical to promote disease modification and preserve quality of life, given the relapsing nature of IMIDs.^5^^,^^6^^,^^7^ Nonetheless, the requirement for invasive, milligram-level antibody injections over extended treatment durations, potentially spanning a lifetime, imposes substantial physiological, psychological, and economic burdens on patients, thereby posing challenges to treatment adherence.^8^^,^^9^^,^^10^^,^^11^

Oral administration, noted for its non-invasive nature and resulting high patient compliance, provides a patient-friendly alternative to injectable antibody formulations with the potential to significantly enhance treatment adherence.^12^^,^^13^^,^^14^^,^^15^ However, the harsh gastrointestinal environment and limited epithelial transport pose substantial obstacles to the oral delivery of antibodies.^12^^,^^16^ While oral delivery systems of antibody-based drugs have been explored and shown promise in addressing these challenges,^17^^,^^18^^,^^19^ many rely on complex manufacturing and exhibit low drug utilization efficiency, resulting in high costs and limited accessibility. Engineered probiotics represent an innovative solution to these challenges. As live microorganisms that confer health benefits when consumed in adequate amounts,^20^ certain probiotic strains have demonstrated anti-inflammatory effects relevant to inflammatory bowel disease (IBD),^21^^,^^22^^,^^23^^,^^24^ a typical IMID. With the advent of synthetic biology, probiotics can now be engineered to produce and deliver therapeutic molecules *in situ*, offering a self-replicating, cost-effective platform for oral biologics.^24^^,^^25^^,^^26^^,^^27^ Some strains also exhibit strong gut persistence and resistance to environmental stress,^28^ making them attractive vehicles for chronic disease therapy. Thus, orally administered engineered probiotics, offering low cost and ease of accessibility, are promisingly associated with improved patient compliance in IMIDs.

In this study, we leverage *Escherichia coli* Nissle 1917, a clinically utilized probiotic strain renowned for its exceptional gut colonization and intrinsic anti-inflammatory properties,^29^ to build an engineered antibody delivery platform. The ease of production and notable capacity for *in situ* protein synthesis make *E. coli* Nissle 1917 a cost-effective and durable alternative to conventional antibody delivery. We develop the AIDEN (aid for IMIDs: engineered EcN) platform, which utilizes engineered *E. coli* Nissle 1917 strains for *in situ* intestinal synthesis and delivery of single-chain variable fragments (scFvs) targeting key mediators in IMIDs. AIDEN strains remain viable and active in the gut and are engineered to respond to orally administered arabinose (Ara), enabling on-demand secretion of therapeutic scFvs for controlled, sustained intervention. As a proof of concept, we construct AIDEN-IL17, an *E. coli* Nissle 1917 variant that secretes anti-IL-17A scFvs. Our findings demonstrate that biweekly oral administration of AIDEN-IL17 in mouse models of psoriasis and IBD effectively reduces systemic IL-17A levels and attenuates disease progression. These results establish AIDEN as a promising oral delivery platform, offering a high-compliance alternative to injectable antibodies for long-term management of IMIDs.

## Results

### Construction of an Ara-responsive AIDEN variant targeting IL-17A: AIDEN-IL17

**Figure 1:**
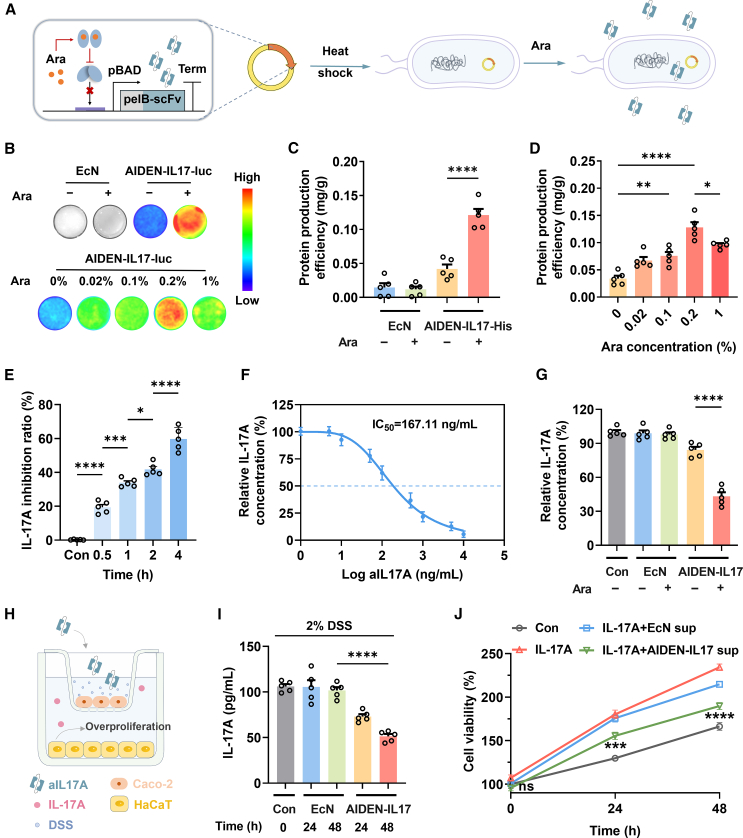
Construction and characterization of AIDEN-IL17 (A) The preparation of AIDEN-IL17 involved the expression mechanism of aIL17A. pBAD, *araBAD* promoter. (B–D) Representative bioluminescence images (B) and bicinchoninic acid assay (BCA) quantification (C and D) of AIDEN-IL17 expressing aIL17A-luc or aIL17A-His under different Ara conditions. See relative data in Figure S1. (E and F) Relative concentration of remaining IL-17A after incubation with aIL17A-His under conditions of different incubation times (E) and aIL17A-His concentrations (F). (G) Relative remaining IL-17A concentration after incubation with *E. coli* Nissle 1917 or AIDEN-IL17 under different Ara conditions. (H) Schematic of the cellular experiment. (I) Remaining IL-17A concentration in the basolateral chamber after different incubation times in DMEM (Con), supernatant from *E. coli* Nissle 1917, or AIDEN-IL17 in the apical chamber. Also see Figure S2. (J) Cell viability of HaCaT in the basolateral chamber after incubation of DMEM (Con), *E. coli* Nissle 1917, or AIDEN-IL17 supernatant (sup) in the apical chamber. Statistical analysis was performed between the IL-17A group and AIDEN-IL17 sup-treated group. Data represent mean ± SEM (*n* = 5). The *p* values in (C), (D), (E), (G), and (I) were determined by one-way ANOVA with Tukey’s multiple comparisons. The *p* values in (J) were determined by two-way ANOVA with Tukey’s multiple comparisons. ns, not significant, ∗*p* < 0.05, ∗∗*p* < 0.01, ∗∗∗*p* < 0.001, and ∗∗∗∗*p* < 0.0001.

IL-17A, a key pro-inflammatory cytokine in IMIDs, has emerged as an effective therapeutic target, with monoclonal antibodies like secukinumab showing clinical success.^30^ Thus, IL-17A was selected as the initial target for development of the AIDEN therapeutic platform, leading to creation of a variant named AIDEN-IL17, secreting scFvs targeting IL-17A (Table S1).^31^ To facilitate controllable synthesis and release of scFvs in *E. coli* Nissle 1917, oral Ara was utilized as a trigger switch, concurrently minimizing unintended expression. On these bases, we constructed a prokaryotic expression plasmid containing an Ara-driven therapeutic circuit, which includes the gene encoding aIL17A with an N-terminal pelB secretion signal, regulated by the *araBAD* promoter. The recombinant plasmid was subsequently introduced into the probiotic strain *E. coli* Nissle 1917, resulting in Ara-responsive AIDEN-IL17 (Figure 1A). To validate the functionality of the Ara-triggered expression system, we generated a reporter strain, AIDEN-IL17-luc, in which the luciferase gene was fused with aIL17A under the *araBAD* promoter. Bioluminescence analysis revealed that AIDEN-IL17-luc exhibited low bioluminescence in the absence of Ara, while luciferase expression was significantly activated in the presence of 0.2% Ara, corresponding to the highest bioluminescence intensity (Figures 1B and S1A). Further confirmation by SDS-PAGE and western blot also demonstrated Ara-inducible expression of AIDEN-IL17 (Figures S1B and S1C). Following His tag purification, quantitative analysis of aIL17A expression substantiated these conclusions (Figures 1C and 1D). In addition, the secretion efficiency was determined to be approximately 10.8%, consistent with earlier research (Figure S1D).^32^ We next assessed the functional activity of purified aIL17A. Binding and neutralization assays showed that extended incubation increased neutralizing capacity, with a half-maximal inhibitory concentration (IC_50_) of 167 ng mL^−1^ at 4 h (Figures 1E and 1F). Importantly, AIDEN-IL17 culture supernatant containing Ara was capable of directly neutralizing IL-17A, demonstrating the ability of the engineered probiotic to secrete biologically active aIL17A (Figure 1G).

To model intestinal delivery and subsequent effect, we used a Transwell co-culture system; Caco-2 cells formed the upper epithelial barrier, and HaCaT keratinocytes were cultured below (Figure 1H). Given that increased intestinal permeability is implicated in IMID pathogenesis,^33^^,^^34^ 2% dextran sodium sulfate (DSS) salt was added to enhance the permeability of the Caco-2 cell monolayers, imitating a permeable intestinal barrier. In untreated Caco-2 monolayers, translocation of aIL17A was minimal (Figure S2). In contrast, DSS-treated monolayers allowed aIL17A to cross into the basolateral chamber, where it effectively neutralized IL-17A in a time-dependent manner (Figure 1I). To evaluate the downstream effect of IL-17A neutralization, we investigated the ability of AIDEN-IL17 to reduce IL-17A-stimulated proliferation of HaCaT cells. IL-17A stimulation significantly enhanced proliferation over time compared to controls (Figure 1J). However, treatment with AIDEN-IL17 culture supernatant, delivered through a permeabilized Caco-2 layer, significantly reduced this response, whereas *E. coli* Nissle 1917 supernatant had no effect. These findings confirmed that AIDEN-IL17-secreted scFvs could traverse a compromised intestinal barrier and functionally block IL-17A-driven cellular responses.

### Evaluation of AIDEN-IL17 functionality and therapeutic potential *in vivo*

**Figure 2:**
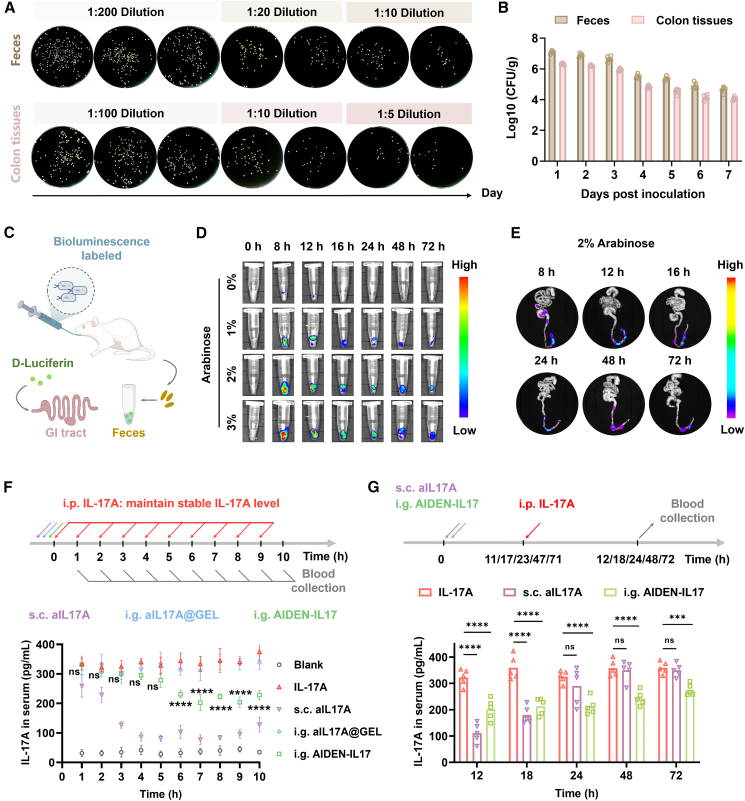
*In vivo* performance of AIDEN-IL17 (A and B) Representative images (A) and quantification (B) of plate colony counting of colon tissues and fecal samples, harvested from mice daily after oral gavage with AIDEN-IL17 (*n* = 5). Also see Figures S3 and S4. (C–E) Experimental design (C) and representative bioluminescence images of feces (D) and colon tissues (E) acquired at the indicated time point post oral gavage (*n* = 5). See quantification in Figure S5. (F) Experimental design and corresponding serum IL-17A levels (*n* = 3, 12 mice per group for rotating collection of blood). Statistical analysis was performed between the IL-17A group and i.g. AIDEN-IL17 group. (G) Experimental design and corresponding serum IL-17A levels 12, 18, 24, 48, and 72 h post gavage (*n* = 5). Data represent mean ± SEM. The *p* values in (F) and (G) were determined by two-way ANOVA with Tukey’s multiple comparisons. ∗*p* < 0.05, ∗∗*p* < 0.01, ∗∗∗*p* < 0.001, and ∗∗∗∗*p* < 0.0001.

To assess the *in vivo* performance of AIDEN-IL17, we first characterized its degradation and clearance kinetics after oral administration. Despite rapid metabolism in the gastrointestinal tract (GIT), the colonic abundance of AIDEN-IL17 stabilized by 24 h post administration and remained at relatively high levels—approximately 0.01% of the total oral dose—through 72 h (Figures 2A, 2B, and S3). Moreover, its gut colonization capacity was comparable to that of the *E. coli* Nissle 1917 chassis, indicating that the genetic modifications did not impair natural competitiveness (Figure S4). Collectively, AIDEN-IL17 exhibited sustained high levels for the initial 3 days post administration, supporting a 3-day dosing interval for subsequent experiments. To further evaluate *in vivo* aIL17A expression, we utilized a luciferase-tagged strain, AIDEN-IL17-luc (Figure 2C). Following gavage with 10^10^ colony-forming units (CFUs) of AIDEN-IL17-luc and oral administration of Ara at various concentrations, fecal samples were collected for bioluminescence analysis. The 2% Ara solution induced the highest expression of aIL17A-luc (Figures 2D and S5A), and this concentration was selected for further *in vivo* testing. Imaging of the GI tract revealed a rapid decline in aIL17A-luc signal during the first 24 h, followed by a stable level over the next 48 h (Figures 2E and S5B). These data confirmed that AIDEN-IL17 can persist in the colon and maintain functional activity for at least 3 days following a single oral dose.

Building on these findings, we explored the potential of AIDEN-IL17 to neutralize excessive systemic IL-17A *in vivo*. Timed intraperitoneal injections were employed to maintain a stable and elevated level of IL-17A in murine serum (approximately 300–400 pg mL^−1^). Leveraging this model, we evaluated the effects of three treatments on serum IL-17A: (1) subcutaneous injection of purified aIL17A (subcutaneous [s.c.] aIL17A), (2) oral administration of aIL17A encapsulated in a protective hydrogel formed by sodium alginate and chitosan (intragastric [i.g.] aIL17A@GEL), and (3) oral administration of AIDEN-IL17 (i.g. AIDEN-IL17). The s.c. aIL17A treatment rapidly and consistently reduced serum IL-17A levels to near-normal for at least 10 h (Figure 2F). Although i.g. administration of AIDEN-IL17 did not exhibit immediate effects within the first 5 h, it subsequently led to a moderate reduction in serum IL-17A levels and demonstrated sustained control during continued IL-17A injection. In contrast, aIL17A@GEL demonstrated minimal efficacy, likely due to poor mucosal absorption and short gut residence time. To further investigate the duration of AIDEN-IL17 activity, we extended the observation period to 72 h, with mice receiving a single IL-17A injection 1 h prior to each time point (Figure 2G). In contrast to s.c. aIL17A, which lost efficacy after 24 h and failed to neutralize excess serum IL-17A, i.g. AIDEN-IL17 retained its neutralizing potential against elevated serum IL-17A for at least 3 days post administration. However, aIL17A was not detected within the ELISA detection limit (Figure S6). We hypothesize that the intact gut barrier limited aIL17A translocation into the circulation *in vivo*, but its high affinity for IL-17A and extended half-life were sufficient to achieve functional neutralization. Together, these findings support the potential of AIDEN-IL17 as an orally administered, long-acting therapeutic platform capable of sustained modulation of systemic IL-17A levels in IMIDs.

### Therapeutic efficacy of AIDEN-IL17 in an IMQ-induced psoriasiform dermatitis model

To assess the therapeutic potential of AIDEN-IL17 in IMIDs, we employed the imiquimod (IMQ)-induced psoriasiform dermatitis model,^35^ which closely mimics clinical psoriasis, a prototypical IMID effectively treated with IL-17A-targeting monoclonal antibodies. Mice were topically treated with IMQ cream to induce skin lesions and then orally administered escalating doses of AIDEN-IL17 (10^7^–10^11^ CFUs) (Figure S7). Notably, a dose-dependent reduction in the psoriasis area and severity index (PASI) score involving erythema, desquamation, and induration was observed. However, increasing the dose beyond 10^10^ CFUs did not yield any further significant therapeutic enhancement; thus, 10^10^ CFU was selected as the optimal dose for subsequent experiments. No notable differences in body weight were noted across all dose groups, indicating favorable tolerability for AIDEN-IL17. Encouraged by these favorable therapeutic effects, we further compared AIDEN-IL17 with anti-IL-17A monoclonal antibodies (mAbs), a first-line therapy for psoriasis (Figure S8). Notably, twice-weekly oral AIDEN-IL17 produced therapeutic outcomes comparable to a single injection of anti-IL-17A mAbs, reducing PASI by 55% versus 77%, respectively. Although a difference remains, these findings highlight the potential of AIDEN-IL17 as an adherence-friendly, non-invasive option for psoriasis therapy.

**Figure 3:**
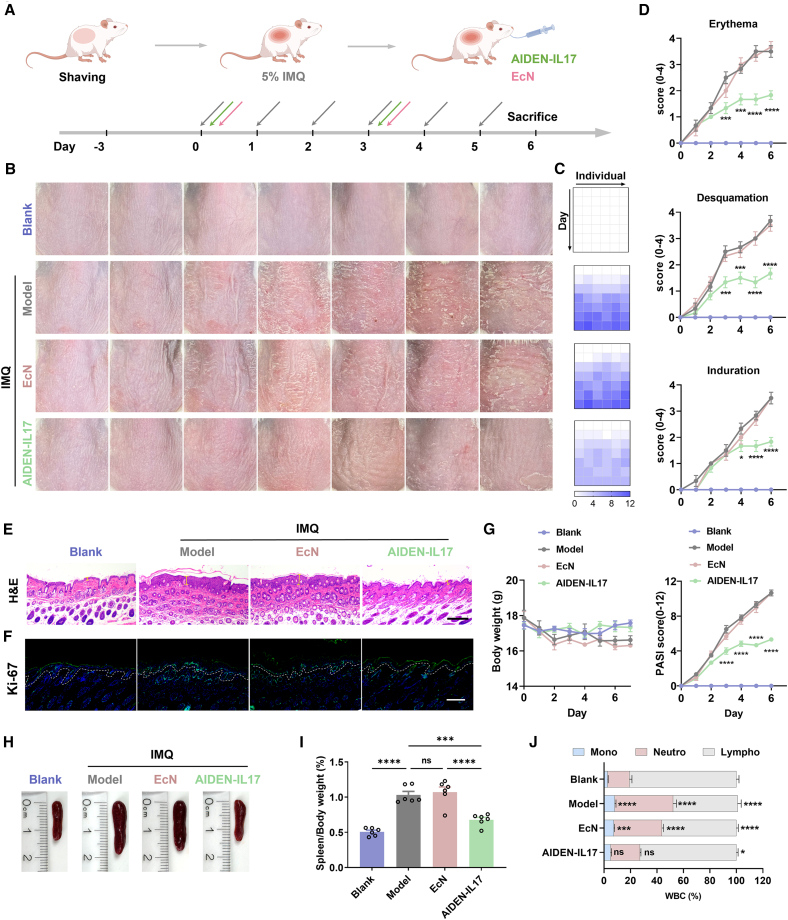
Therapeutic efficacy of AIDEN-IL17 in psoriasiform dermatitis mice (A) Schematic of the experimental timeline. (B) Representative bright-field images of dorsal skins of mice in different groups from days 0–6. (C) Heatmap of the total PASI scores of each mouse from days 0–6. (D) Erythema, desquamation, induration, and total PASI scores each day. Comparisons were made between the model group and AIDEN-IL17 group. (E) Representative images of H&E staining of psoriasis skin. Scale bar, 200 μm. Yellow labeling highlights the epidermis thickness. Also see Figure S9A. (F) Representative images of Ki-67 immunofluorescence staining of psoriasis skin. Scale bar, 100 μm. Also see Figure S9B. (G) Body weight changes of mice. (H and I) Representative bright-field images (H) and corresponding spleen indexes (I). (J) Monocyte, neutrophil, and lymphocyte percentage in total WBCs. Comparisons were made between the blank group and other treatment groups. Also see Figure S9C. Data represent mean ± SEM (*n* = 6). The *p* values in (D) were determined by two-way ANOVA with Tukey’s multiple comparisons. The *p* values in (I) and (J) were determined by one-way ANOVA with Tukey’s multiple comparisons. ∗*p* < 0.05, ∗∗*p* < 0.01, ∗∗∗*p* < 0.001, and ∗∗∗∗*p* < 0.0001.

To elucidate whether the observed therapeutic effect was attributable to the probiotic itself or its secreted substance aIL17A, IMQ mice were treated with either AIDEN-IL17 or *E. coli* Nissle 1917 (Figure 3A). The PASI scores were evaluated daily (Figures 3B–3D). While IMQ treatment alone induced robust psoriasiform inflammation, only AIDEN-IL17 significantly reduced disease severity. Hematoxylin and eosin (H&E) staining of IMQ-induced lesional skin revealed characteristic epidermal thickening and acanthosis (Figures 3E and S9A), which was further confirmed by increased Ki-67 immunofluorescence (Figures 3F and S9B). In contrast, AIDEN-IL17 treatment markedly reduced epidermal thickness and Ki-67 expression, indicating suppressed keratinocyte proliferation. No obvious body weight loss was recorded in any group during the treatment period, suggesting a favorable safety profile of AIDEN-IL17 for future clinical translation (Figure 3G). Since splenomegaly in IMQ-induced mice primarily reflects systemic immune activation and inflammation,^36^ the spleen size and index were assessed. The results showed increases in both parameters in the IMQ group, which were markedly reduced following AIDEN-IL17 treatment, indicating effective suppression of systemic inflammation (Figures 3H and 3I). In addition, while white blood cell counts did not exhibit significant differences, IMQ induction resulted in a decrease in lymphocytes and an increase in monocytes and neutrophils (Figures 3J and S9C). Notably, AIDEN-IL17 effectively reversed these alterations, restoring immune cell levels to those akin to healthy mice. These results demonstrated that AIDEN-IL17 exerts significant control over systemic inflammation and re-establishes immune homeostasis.

### AIDEN-IL17 attenuates psoriasis progression via modulation of IL-17A

**Figure 4:**
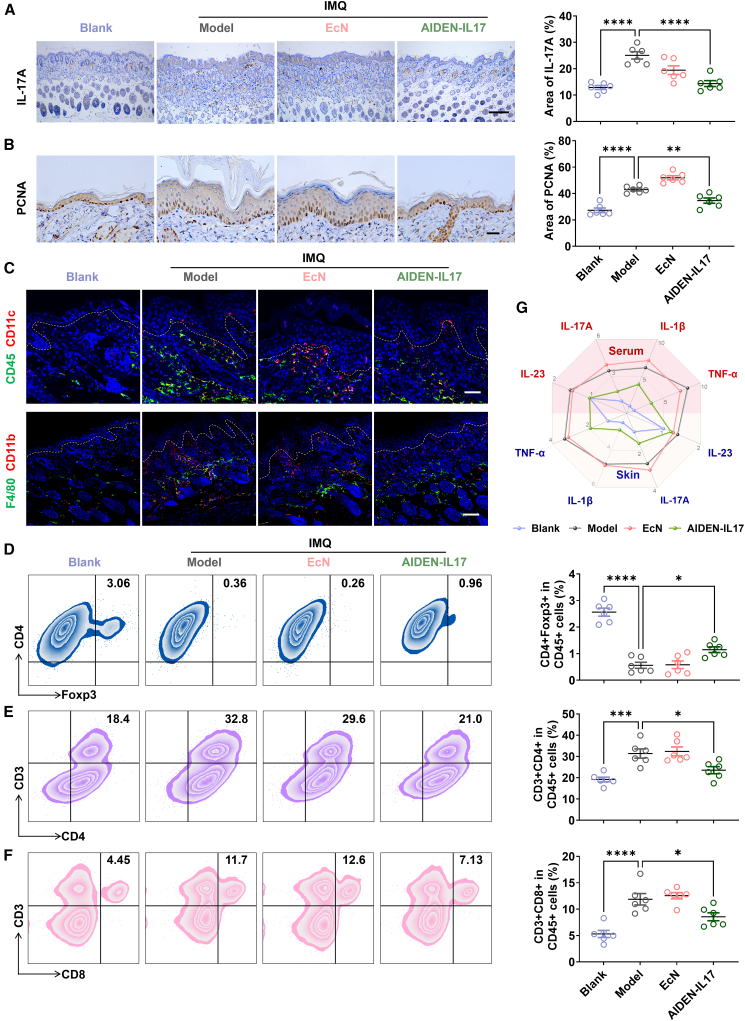
AIDEN-IL17 attenuates psoriasis progression via IL-17A modulation (A and B) Representative images and corresponding quantitative results of IHC staining showing IL-17A (A) and PCNA (B) levels in skin tissues. Scale bar for IL-17A, 200 μm. Scale bar for PCNA, 50 μm. (C) Representative immunofluorescence images of dendritic cells (CD45^+^CD11c^+^) and macrophages (F4/80^+^CD11b^+^) in skins. Scale bar, 50 μm. (D–F) Representative flow cytometry charts and corresponding quantitative results of CD45^+^CD4^+^Foxp3^+^ Treg cells, CD45^+^CD3^+^CD4^+^ T cells, and CD45^+^CD3^+^CD8^+^ T cells. (G) Relative cytokine concentrations in the psoriasis lesion and serum determined by ELISA. Data represent mean ± SEM (*n* = 6). The *p* values in (A), (B), and (D)–(F) were determined by one-way ANOVA with Tukey’s multiple comparisons. ∗*p* < 0.05, ∗∗*p* < 0.01, ∗∗∗*p* < 0.001, and ∗∗∗∗*p* < 0.0001.

We next sought to elucidate whether the therapeutic efficacy of AIDEN-IL17 in mice with IMQ-induced psoriasis is mediated through IL-17A inhibition. In psoriasis, various stimuli activate innate immune cells such as dendritic cells and macrophages, leading to release of IL-23, which subsequently activates Th17 cells to produce IL-17A, ultimately leading to keratinocyte hyperproliferation.^37^ This well-established pathogenic cascade highlights the central role of IL-17A in development and progression of psoriasis. Initially, focusing on the local skin environment, we validated that AIDEN-IL17 effectively suppresses the overproduction of IL-17A within psoriatic skin lesions (Figure 4A), thereby inhibiting IL-17A-induced aberrant keratinocyte proliferation, as evidenced by proliferating cell nuclear antigen (PCNA) immunohistochemistry (IHC) staining (Figure 4B). This direct inhibition of IL-17A at the inflammatory site suggested that AIDEN-IL17 can disrupt the positive feedback loop driving psoriatic pathology. Recognizing that IL-17A neutralization can ameliorate the dysregulated immune microenvironment, we assessed the infiltration of innate immune cells, specifically CD45^+^CD11c^+^ dendritic cells and F4/80^+^CD11b^+^ macrophages, into the skin (Figure 4C). AIDEN-IL17 treatment resulted in a significant reduction in the infiltration of both dendritic cells and macrophages, indicative of effective disease modulation. The decreased infiltration of these antigen-presenting cells further supported the notion that AIDEN-IL17 effectively dampens the inflammatory response in the skin.

Furthermore, to delineate the impact of AIDEN-IL17 on systemic inflammatory conditions, flow cytometry analysis was performed on splenocytes to characterize systemic immune cell populations. The induction of IMQ led to a marked decrease in the proportion of splenic CD4^+^Foxp3^+^ regulatory T (Treg) cells, an effect that was modestly but significantly reversed by AIDEN-IL17 treatment (Figure 4D). The partial restoration of Treg cells, which is crucial for maintaining immune homeostasis, suggested the potential of AIDEN-IL17 to promote immune regulation and mitigate excessive inflammation. Conversely, the elevated frequencies of both CD45^+^CD3^+^CD4^+^ and CD45^+^CD3^+^CD8^+^ T cells observed in the spleen of IMQ-treated mice were effectively reduced by AIDEN-IL17 administration (Figures 4E and 4F). These results suggested that AIDEN-IL17 can modulate the broader systemic immune response, as indicated by changes in immune cell populations within the spleen, a pivotal organ for systemic immune regulation. In addition, the expression levels of the proinflammatory cytokines TNF-α, IL-1, IL-17A, and IL-23 were markedly elevated in IMQ mice but greatly downregulated by AIDEN-IL17 treatment in both skin and serum samples, suggesting effective alleviation of inflammation at both local and systemic levels (Figure 4G). Taken together, these findings provide compelling evidence that AIDEN-IL17 exerts its therapeutic effects in IMQ-induced psoriasiform dermatitis through suppression of IL-17A-relevant innate and adaptive immune responses, effectively inhibiting disease progression.

### Therapeutic efficacy of AIDEN-IL17 in DSS-induced colitis mice

**Figure 5:**
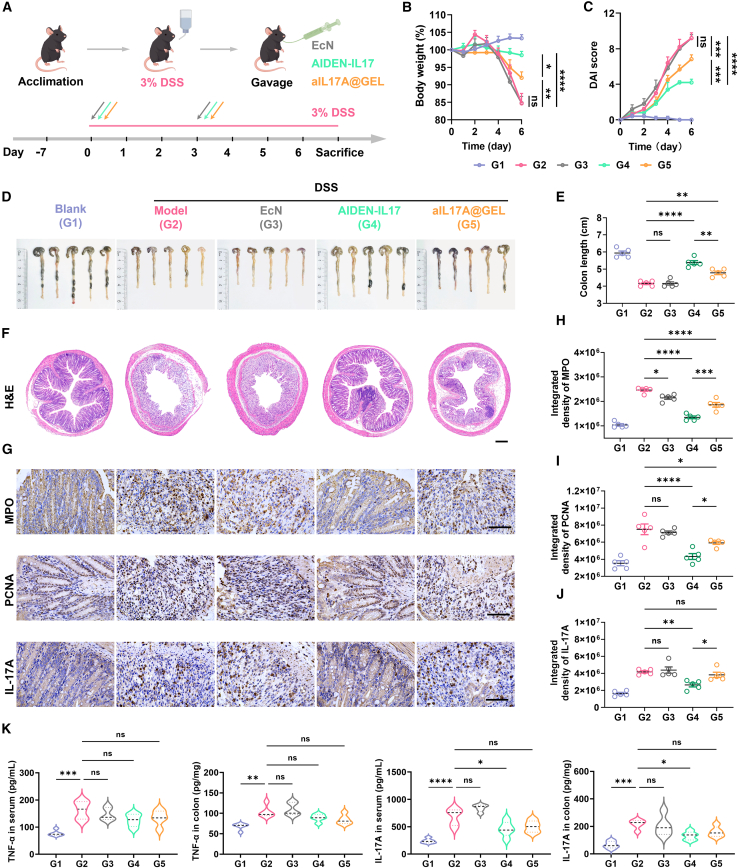
Therapeutic efficacy of AIDEN-IL17 in IBD mice (A) Schematic of the experimental timeline. (B and C) Body weight (B) and DAI score (C) changes of mice in different groups. (D and E) Bright-field images of the colon (D) and corresponding quantification of colon length (E). (F) Representative H&E-stained images of colon tissues. Scale bar, 500 μm. (G–J) Representative images (G) and corresponding quantification of IHC staining of MPO (H), PCNA (I), and IL-17A (J) levels in colon tissues. Scale bar, 50 μm. (K) Cytokine concentrations of TNF-α and IL-17A in colon tissues and serum. Data represent mean ± SEM (*n* = 5). The *p* values in (B) and (C) were determined by two-way ANOVA with Tukey’s multiple comparisons. The *p* values in (E) and (H)–(K) were determined by one-way ANOVA with Tukey’s multiple comparisons. ∗*p* < 0.05, ∗∗*p* < 0.01, ∗∗∗*p* < 0.001, and ∗∗∗∗*p* < 0.0001.

IBD, similar to psoriasis, is another chronic IMID characterized by dysregulated immune responses involving TNF-α and IL-17 signaling pathways. To investigate the therapeutic potential of AIDEN-IL17, C57BL/6 mice were given 3% DSS in drinking water for 7 consecutive days to induce acute IBD. The treatment protocol involved oral administration on days 0 and 3 with either *E. coli* Nissle 1917, AIDEN-IL17, or aIL17A@GEL (Figure 5A). Body weight loss, a key indicator of disease severity, became apparent in untreated mice by day 4. In contrast, mice treated with AIDEN-IL17 maintained stable body weight, while those receiving *E. coli* Nissle 1917 exhibited no protection (Figures 5B and 5C). These trends were reflected in the Disease Activity Index (DAI), supporting the efficacy of AIDEN-IL17 in reducing disease burden. Although aIL17A@GEL showed moderate efficacy, its therapeutic benefit was inferior to that of AIDEN-IL17 and required higher doses and more frequent administration, raising practical limitations for clinical application. On day 7, mice were euthanized for tissue and blood analyses. Notably, colons from AIDEN-IL17-treated mice were significantly longer, by approximately 1.1 cm, than those from DSS-treated mice, consistent with reduced inflammation (Figures 5D and 5E). H&E staining revealed extensive epithelial damage, mucosal necrosis, and lymphocyte infiltration in DSS-treated mice (Figure 5F). In contrast, AIDEN-IL17 markedly preserved colonic architecture and reduced inflammatory damage. To further assess tissue pathology, we evaluated myeloperoxidase (MPO), a marker of inflammation, and PCNA, a marker of tissue regeneration. Both markers were elevated in DSS-treated mice, reflecting active colitis with simultaneous injury and repair (Figures 5G–5I). AIDEN-IL17 significantly attenuated this dual elevation, indicating reduced inflammation and restoration of mucosal homeostasis. Notably, AIDEN-IL17 significantly decreased the IL-17A levels in colon tissues, demonstrating that efficient neutralization of IL-17A contributed to the inflammation alleviation (Figures 5G and 5J). Furthermore, the levels of TNF-α and IL-17A in serum and colon tissues were measured (Figure 5K). The TNF-α levels, which showed no statistically significant differences between all treated groups and the model group in both serum and colon tissues, suggested an upstream regulatory role in the initial stage of IBD pathogenesis. The level of IL-17A in the AIDEN-IL17 group was significantly lower than in the model group, while no significant differences were observed between other treatment groups and the model group. Taken together, these results demonstrate that AIDEN-IL17 effectively alleviates intestinal inflammation by locally neutralizing IL-17A, thereby mitigating IL-17A-mediated tissue damage and restoring epithelial integrity.

### Toxicological evaluation of AIDEN-IL17

**Figure 6:**
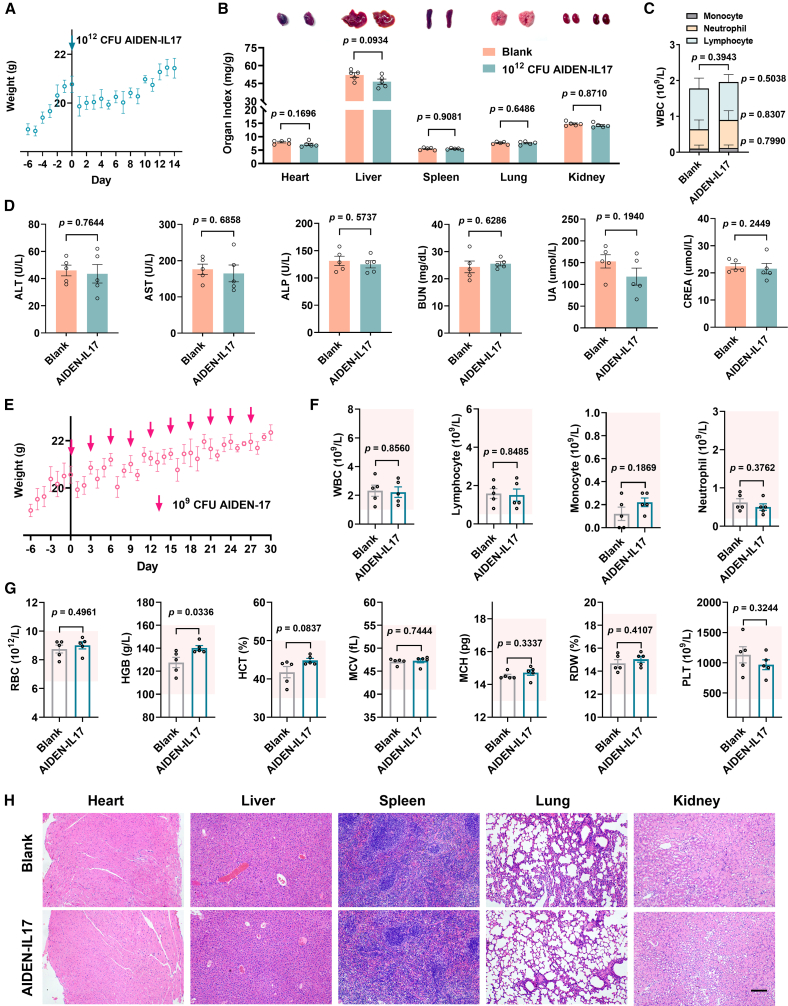
Toxicological evaluation of AIDEN-IL17 (A) The body weight changes within 14 days in acute toxicity studies. (B) Representative bright-field images and corresponding organ indexes. See representative H&E-stained sections of main organs in Figure S10A. (C) Monocyte, neutrophil, and lymphocyte counts. (D) Representative blood biochemistry parameters. ALT, alanine aminotransferase; AST, aspartate aminotransferase; ALP, alkaline phosphatase; BUN, blood urea nitrogen; UA, uric acid; CREA, creatinine. Also see Figure S10B. (E) The body weight changes in the long-term toxicity studies. AIDEN-IL17 was administered once every 3 days. (F and G) Routine blood examination. RBC, red blood cell; HGB, hemoglobin; HCT, hematocrit; MCV, mean corpuscular volume; MCH, mean corpuscular hemoglobin; RDW, red cell distribution width; and PLT, platelet. (H) Representative H&E-stained sections of main organs. Scale bar, 100 μm. Data represent mean ± SEM (*n* = 5). The *p* values in (B)–(D) and (F) were determined by two-tailed Student’s *t* test. ∗*p* < 0.05, ∗∗*p* < 0.01, ∗∗∗*p* < 0.001, and ∗∗∗∗*p* < 0.0001.

To assess the safety of AIDEN-IL17, both acute and long-term toxicological studies were conducted in healthy mice. For acute toxicity analysis, healthy mice received a single oral gavage of AIDEN-IL17 at an excessive dose of 10^12^ CFUs per mouse on day 0, which was 100 times the therapeutic dose. A temporary reduction in body weight was observed in the initial days following administration of this high dose, with subsequent recovery of body weight and no mortality recorded during the monitoring period (Figure 6A). On day 14, the mice were euthanized for necropsy and blood analysis. Organ coefficients for the heart, liver, spleen, lungs, and kidneys showed no statistically significant differences between the AIDEN-IL17 and control groups (Figure 6B). As organ coefficients are sensitive indicators of organ hypertrophy or toxicity, these results suggested no overt organ damage. Histological examination using H&E staining corroborated the absence of pathological abnormalities in these organs (Figure S10A). Additionally, hematological assessments revealed no significant differences in neutrophil, lymphocyte, or total white blood cell counts between control and AIDEN-IL17-treated animals, indicating no disruption of normal hematopoiesis (Figure 6C). Furthermore, serum levels of alanine aminotransferase (ALT), aspartate aminotransferase (AST), alkaline phosphatase (ALP), uric acid (UA), creatinine (CREA), blood urea nitrogen (BUN), γ-glutamyl transpeptidase (γ-GT), albumin (ALB), total bilirubin (TBIL), and direct bilirubin (DBIL), key indicators of liver and kidney function, showed no significant differences between the groups (Figures 6D and S10B), further supporting the conclusion that AIDEN-IL17 induced no significant acute toxicity. Collectively, these findings demonstrate that AIDEN-IL17 does not induce acute toxicity, even at doses far exceeding therapeutic levels.

To evaluate long-term safety, healthy mice were orally administered 10^9^ CFUs of AIDEN-IL17 via gavage every 3 days for a duration of 1 month, simulating chronic exposure. Although a slight and transient decrease in body weight was observed the day following each gavage, the mice quickly adapted, and the weight fluctuations became negligible after the initial few administrations (Figure 6E). Importantly, no mortality was observed throughout the study period. Considering the possibility that prolonged IL-17A blockade could lead to hematological alterations such as leukopenia, thrombocytopenia, and anemia, a complete blood count (CBC) was conducted to evaluate the impact of AIDEN-IL17 on these parameters. There were no statistically significant differences in any of the assessed hematological parameters, including white blood cell (WBC) count, red blood cell (RBC) count, hemoglobin (HGB), hematocrit (HCT), mean corpuscular volume (MCV), mean corpuscular HGB (MCH), red cell distribution width (RDW), and platelet (PLT) count, between the AIDEN-IL17-treated group and the control group, with all values within the normal reference range (Figures 6F and 6G). Additionally, H&E-stained sections of major organs, including the heart, liver, spleen, lungs, and kidneys, revealed no overt pathological abnormalities or signs of tissue damage (Figure 6H). These findings indicated that AIDEN-IL17 exhibits a promising long-term safety profile in mice, laying the groundwork for clinical use.

## Discussion

Developing oral antibody drugs has long been an aspirational goal, especially for chronic diseases like IMIDs. To achieve valid antibody delivery through the complex GIT and mucus-covered cellular barrier, recent efforts involving protein design, modification, encapsulation, and delivery system innovation have begun to yield promising progress. Using protein design strategies, a *de novo*-designed miniprotein exhibited preclinical activity when orally administered in mice, and an engineered protease-stable single-domain antibody enabled oral delivery.^12^^,^^14^ Regarding protein modification and encapsulation, a transmucosal carrier, fluorocarbon-modified chitosan, helped oral antibodies achieve effective bioavailability in mice.^17^ Through innovation in oral delivery systems, a gastric auto-injector was developed and achieved milligram-level antibody delivery into the blood.^38^ Although these therapies are more convenient than parenterally administered antibodies, their high cost of manufacturing and safety risks are significant downsides. Engineered probiotics offer an accessible and inexpensive alternative.

Using *E. coli* Nissle 1917 as the chassis probiotic, we developed AIDEN-IL17, a variant of the AIDEN platform engineered for *in situ* delivery of aIL17A, as a proof of concept. Leveraging the colonization properties of the chassis, a single administration of AIDEN-IL17 enables sustained intestinal antibody delivery for up to 3 days. While subcutaneous injection of aIL17A provides a more rapid and extensive IL-17A blockade, AIDEN-IL17 demonstrated notably prolonged efficacy. Specifically, moderate IL-17A neutralization was still observed 72 h post a single oral dose. Beyond efficacy, engineered probiotics provide distinct practical advantages. Unlike injectable antibodies, which require stringent purification, cold-chain storage, and sterile administration, probiotic-based therapies can be prepared at lower cost, distributed with relative ease, and administered orally without professional supervision. These features align closely with the needs of patients managing chronic IMIDs, where long-term adherence is critical. Importantly, AIDEN-IL17 exhibited no detectable toxicity, even with high-dose or prolonged administration, supporting its safety profile for chronic use. Collectively, our findings introduce a promising oral alternative to injectable antibodies for managing chronic IMIDs, leveraging an engineered probiotic platform that enables *in situ* intestinal antibody production and delivery. The platform, AIDEN, shows potential as a versatile oral delivery system for IMID treatment and could be adapted for other therapeutic antibodies, such as those targeting IL-23 or TNF, to treat a wider range of IMIDs.

### Limitations of the study

The potential impact of sex on the study findings is acknowledged as a limitation, as independent sex-specific analyses were not conducted across the experimental mouse models. Even a single-chain antibody component, which constitutes approximately one-sixth of a full-length antibody, is still recognized by the intestinal epithelial barrier as a molecule that poses considerable challenges for traversal. Thus, enhancing the efficiency of transmucosal delivery remains imperative. Future optimization of the platform should prioritize the identification of more suitable and potent antibodies through advances in protein design (e.g., IL-23-binding mini-proteins); enhancement of transmucosal delivery mechanisms (e.g., utilizing cell-penetrating peptides, Fc fragments, or transport enhancers); or employment of immune-evasive bacterial outer membrane vesicles. These strategies aim to reduce intestinal degradation, improve systemic drug concentrations, extend dosing intervals, and ultimately enhance patient compliance.

## Resource availability

### Lead contact

Requests for further information, resources, and reagents should be directed to and will be fulfilled by the lead contact, Zhongliang Wang (wangzl@xidian.edu.cn).

### Materials availability

All unique/stable reagents generated in this study are available from the lead contact with a completed materials transfer agreement.

### Data and code availability

• All data reported in this paper will be shared by the lead contact upon request.
• Any additional information required to reanalyze the data reported in this paper is available from the lead contact upon request.
• No custom computer code was generated or used in this study.

## Acknowledgments

We sincerely acknowledge Prof. Wei Wei for mentorship and support, which were instrumental in the completion of this research. This work was financially supported by the 10.13039/501100012166National Key Research and Development Program of China (2024YFC2421000 and 2022YFB3203800), the 10.13039/501100001809National Natural Science Foundation of China (W2411083, 32401188, and 82572430), the 10.13039/501100002858China Postdoctoral Science Foundation (2025T180787), the 10.13039/501100015401Key Research and Development Program of Shaanxi (2024SF-YBXM-126), the 10.13039/501100021171Guangdong Basic and Applied Basic Research Foundation (2023A1515111147), the Xi'an Association for Science and Technology Youth Talent Support Program (0959202513018), the 10.13039/501100012226Fundamental Research Funds for the Central Universities (ZYTS25094 and QTZX25107), and the Xidian University Specially Funded Project for Interdisciplinary Exploration (TZJH2024041). We thank the Instrumental Analysis Center of Xidian University for providing test equipment and the Comprehensive Experimental Center for chemistry and bioscience.

## Author contributions

Z.W. and C.Q. conceived and supervised the project. Z.W., C.Q., and S.S designed the experiments. J.L., L.W., B.P., Y.S., Y.C., and R.Z. performed all experiments. All authors analyzed and discussed the data. C.Q., S.S., and Z.W. wrote the paper.

## Declaration of interests

The authors declare no competing interests.

## STAR★Methods

### Key resources table

**Table:** 

REAGENT or RESOURCE	SOURCE	IDENTIFIER
**Antibodies**		

APC-*anti*-mouse CD8 Antibody	BioLegend	RRID: AB_312750
PE/Cyanine7-*anti*-mouse CD4 Antibody	BioLegend	RRID: AB_312706
FITC-*anti*-mouse CD3 Antibody	BioLegend	RRID: AB_312660
PE-*anti*-mouse CD45 Antibody	BioLegend	RRID: AB_2563597
Alexa Fluor 488-*anti*-mouse Foxp3 Antibody	BioLegend	RRID: AB_439747
Ki-67 polyclonal antibody	Wuhan Servicebio Biotechnology	RRID: AB_2918145
F4/80 polyclonal antibody	Wuhan Servicebio Biotechnology	RRID: AB_2881149
CD11b polyclonal antibody	Wuhan Servicebio Biotechnology	RRID: AB_3670104
CD11c polyclonal antibody	Wuhan Servicebio Biotechnology	RRID: AB_2805960

**Bacterial and virus strains**		

*Escherichia coli* Nissle 1917	Fenghui Biotechnology	sc2023102687

**Chemicals, peptides, and recombinant proteins**		

D-Luciferin potassium salt	Adamas	115144-35-9
Alginic acid sodium salt	Adamas	9005-38-3
Chitosan	Macklin	9012-76-4
Recombinant Mouse IL-17A	BioLegend	576006
Imiquimod cream	Sichuan Mingxin Pharmaceutical	H20030129
L–(+)–Arabinose	Beyotime	ST1420
Dextran sulfate sodium salt	Yesen Biological Technology	60316ES60
Anti-mouse IL-17A-InVivo	Selleck China	A2120
His-tag protein purification beads	Beaver Biotechnology	70501–5
Dulbecco’s modified eagle medium (DMEM)	Gibco	C11995500BT
Fetal bovine serum (FBS)	Gibco	10270–106
Penicillin/streptomycin (PS)	Gibco	15140122

**Critical commercial assays**		

BCA Protein Assay kit	Beyotime	P0012
Cell Counting Kit-8	Beyotime	C0037
Mouse TNF-α ELISA kit	Beyotime	PT512
Mouse IL-1β ELISA kit	Beyotime	PI301
Mouse IL-17A ELISA kit	Beyotime	PI545
Mouse IL-23 ELISA kit	Beyotime	PI655

**Experimental models: Cell lines**		

HaCaT cell	Shanghai Zhongqiao Xinzhou	ZQ0044
Caco-2 cell	Shanghai Zhongqiao Xinzhou	ZQ0056

**Experimental models: Organisms/strains**		

Balb/c mice	Chongqing Tengxin	N/A
C57BL/6 mice	Chongqing Tengxin	N/A

**Recombinant DNA**		

pelB-aIL17A-His in pBAD33	This Study	N/A
pelB-aIL17A-luc in pBAD33	This Study	N/A
pelB-aIL17A-HA in pBAD33	This Study	N/A
pelB-aIL17A in pBAD33	This Study	N/A

**Software and algorithms**		

GraphPad Prism 9	GraphPad	9.5.0
ImageJ	https://imagej.net/ij/	1.8.0

### Experimental model and study participant details

#### Cell types and culture conditions

The HaCaT cells (ZQ0044) and Caco-2 cells (ZQ0056) were obtained from Shanghai Zhongqiao Xinzhou (Shanghai China). Both were proved to be free of mycoplasma contamination and were authenticated by short tandem repeat (STR) profiling. The HaCaT cells and Caco-2 cells were cultured in the dulbecco’s modified eagle medium (DMEM, Gibco), containing 10% fetal bovine serum (FBS, Gibco) and 1% penicillin/streptomycin (PS, Gibco). The cells were incubated at 37°C with 5% CO_2_.

#### Bacteria strains and culture conditions

*Escherichia coli* Nissle 1917 was obtained from Fenghui Biotechnology (China). All the other bacteria were engineered from *Escherichia coli* Nissle 1917. The bacteria were grown at 37°C in Luria-Bertani (LB) medium.

#### Animal model and care

Animal protocols related to this study were reviewed and approved by the Institutional Animal Care and Use Committee of Air Force Medical University (approval number: 20230463). Six-week-old female Balb/c were used for IMQ-induced psoriasis mice model and eight-week-old female C57BL/6 mice were used for DSS-induced IBD mice model. Male Balb/c at 8 weeks were used for other *in vivo* experiments, considering their generally higher tolerance for repeated blood collection procedures. All the mice were sourced from Chongqing Tengxin Co., Ltd and housed under controlled environmental conditions with a temperature range of 20°C–26°C, a relative humidity of 30–70%, and a 12-h light-dark cycle.

### Method details

#### Preparation of engineered bacteria AIDEN-IL17

The IL-17A scFvs gene sequence was obtained from a previously reported study.^39^ We fused the aIL17A gene with an N-terminal pelB signal sequence to the pBAD promoter in pBAD33 plasmid, generating the Ara-reactive recombinant plasmid pBAD33-aIL17A for aIL17A expression. In addition, to facilitate subsequent purification or detection, His-tag or luciferase gene sequence was added following aIL17A sequence to construct pBAD33-aIL17A-His or pBAD33-aIL17A-luc. The designed plasmids were synthesized by BGI (HuaDa Gene). These plasmids were then, respectively, introduced into EcN via heat shock method, resulting in the Ara-responsive strains AIDEN-IL17 and so on.

#### Ara-responsive performance of AIDEN-IL17

To evaluate the specificity and sensitivity of the Ara response, AIDEN-IL17-luc with the pBAD33-aIL17A-luc plasmid was employed for visual representation for bioluminescence imaging analysis, while AIDEN-IL17-His with the pBAD33-aIL17A-His plasmid was for quantification analysis by BCA. The wild type strain was as the control, termed as EcN. Following experimental groups were established to assess the Ara-dependence: EcN without Ara, EcN with 0.2% Ara, AIDEN-IL17-luc without Ara and AIDEN-IL17 with 0.02%, 0.1%, 0.2% or 1% Ara. After a 6-h incubation under the respective conditions, bacterial pellets of 10^9^ CFU were suspended in 400 μL 15 mg·mL^−1^ D-luciferin substrate solutions in a 24-well plate and imaged. For quantification, the same groups of AIDEN-IL17-His were collected and sonicated, and corresponding aIL17A-His was purified by His-tag protein purification beads for BCA assay.

#### IL-17A neutralizing performance of AIDEN-IL17

To assess the IL-17A-neutralization ability of AIDEN-IL17, aIL17A-His protein was extracted via His-tag protein purification beads and concentrated by ultrafiltration. The ultrafiltered aIL17A-His solution was adjusted to concentration of 100 ng·mL^−1^, and 10 μL was added to individual tubes pre-loaded with 100 μL of 1 ng·mL^−1^ IL-17A. As a control, 10 μL of solution without aIL17A-His was added to IL-17A. Reactions were incubated at 37°C for 0.5, 1, 2 or 4 h, after which the remaining IL-17A concentrations were measured by IL-17A ELISA. To depict the inhibition curve, aIL17A-His solutions ranging from 10^0^ to 10^4^ ng·mL^−1^ were prepared, and 10 μL of each concentration was added into 100 μL of 1 ng·mL^−1^ IL-17A. Following a 4-h incubation at 37°C, the IL-17A concentrations were determined by IL-17A ELISA. To directly evaluate the IL-17A-neutralizing ability of AIDEN-IL17 itself, 10^10^ CFU of EcN or AIDEN-IL17 in the logarithmic growth phase were collected, induced with 0.2% Ara in DMEM containing 1 ng·mL^−1^ IL-17A, and incubated at 37°C for 6 h. Subsequently, the IL-17A concentrations in the mixtures were measured by IL-17A ELISA.

#### Evaluation of aIL17A performance at cellular level

To assess the performance of aIL17A at the cellular level, Caco-2 cells were cultured in 24-well transwell chambers for 21 days to establish a monolayer intestinal epithelial cell barrier. On day 22, the apical chambers, in DSS+ groups, were treated with medium containing 2% DSS for 48 h to induce permeability. The DSS– group was not treated with DSS. On day 24, culture supernatants from 0.2% Ara-induced EcN or AIDEN-IL17 were added to the apical chambers, and the basolateral chambers received DMEM supplemented with 100 pg·mL^−1^ IL-17A. After 12, 24, or 48 h, IL-17A levels in the basolateral chambers were quantified by ELISA. In a separate experiment using the same transwell model, HaCaT cells were seeded in the basolateral chambers at a density of 2×10^5^ cells/well on day 23. On day 24, either EcN or AIDEN-IL17 culture supernatants, or DMEM (as control), were added to the apical chambers. The basolateral medium was then replaced with DMEM containing 100 pg·mL^−1^ IL-17A, except for the control group, where apical chambers received DMEM alone and basolateral chambers received IL-17A-free DMEM. HaCaT cell viability in the basolateral chambers was then measured using a CCK-8 assay after 0, 24, or 48 h.

#### Colonization potential of AIDEN-IL17 in mice

To investigate the *in vivo* colonization of AIDEN-IL17, male Balb/c at 8 weeks of age were randomly assigned to seven groups (*n* = 5 per group) and administered with 10^10^ CFU of AIDEN-IL17, or EcN as a control, by oral gavage. Weighed fecal samples and colon tissue (1 cm proximal to the anus) were collected from each group at 4, 8, 12-h, on 1, 2, 3, 4, 5, 6, and 7-day post-gavages. The collected tissues were then homogenized on ice and subjected to serial dilutions before being plated for colony enumeration. Following a 12-h incubation at 37°C, colony counts were determined.

#### Ara concentration optimization *in vivo*

To optimize the Ara induction concentration *in vivo*, male Balb/c at 8 weeks of age were administered 10^10^ CFU of AIDEN-IL17-luc by oral gavage, followed by the addition of 0%, 1%, 2%, or 3% Ara to their drinking water (*n* = 5 per group). Fecal samples were collected at 0, 8, 12, 16, 24, 48, and 72-h post induction and subjected to bioluminescence imaging after the addition of 500 μL of 15 mg·mL^−1^ D-luciferin substrates.

#### Synthesis and distribution of aIL17A in mice

8 weeks old male Balb/c mice were orally administered 10^10^ CFU of AIDEN-IL17, followed by the provision of 2% Ara in their drinking water. The mice were euthanized at 8, 12, 16, 24, 48, or 72 h after administration, and the entire GI tract, from stomach to colon, was isolated. The GI tract was then infused with 2 mL of 15 mg·mL^−1^ D-luciferin potassium solution for bioluminescence analysis.

#### Synthesis of aIL17A@GEL

aIL17A@GEL was prepared according to a classical synthesis method. Briefly, sodium alginate (30 mg) was dissolved in 1 mL deionized water and thoroughly stirred to ensure complete dissolution. Then, 1 mg of aIL17A was added and gently stirred for 10 min. This mixture was then dropped into a calcium chloride solution (20 mg·mL^−1^) under constant stirring at 27°C for 10 min to form alginate beads. After washing with deionized water three times, the beads were incubated in a chitosan solution (30 mg·mL^−1^) at 25°C with gentle shaking for 60 min. Finally, the beads were washed three times with deionized water to obtain aIL17A@GEL.

#### IL-17A-neutralizing ability of AIDEN-IL17 in mice

To assess the *in vivo* IL-17A-antagonizing ability of AIDEN-IL17, male Balb/c mice (8 weeks old) were randomly divided into five groups (*n* = 12). To maintain IL-17A concentrations between 300 and 400 pg·mL^−1^, mice received intraperitoneal injections of 0.1 μg (100 μL) IL-17A at 0, 2, 4, 6, and 8 h, alternating with 0.05 μg (50 μL) IL-17A at 1, 3, 5, 7, and 9 h. At 0 h, the groups were respectively treated as follows: subcutaneous injection of aIL17A (2 μg, 200 μL), oral gavage of aIL17A@GEL (5 μg, 200 μL), and oral gavage of 10^10^ CFU AIDEN-IL17 with 2% Ara in drinking water. Blood samples were then collected from the submandibular vein hourly (4-h rotation) and analyzed for IL-17A levels by ELISA. To further evaluate the long-term IL-17A-controlling effect of AIDEN-IL17, male Balb/c mice were randomly divided into three groups (*n* = 10 per group). The experimental protocol was modified as follows: At 0 h, mice received either a subcutaneous injection of aIL17A (2 μg, 200 μL) or an oral gavage of 10^10^ CFU AIDEN-IL17 with 2% Ara in drinking water. Blood samples were collected at 12, 18, 24, 48, and 72 h for IL-17A analysis. One hour prior to each blood collection, mice received an intraperitoneal injection of 0.1 μg (100 μL) IL-17A.

#### Establishment of IMQ-induced psoriasiform dermatitis mouse model and *in vivo* treatment

Female Balb/c mice (6 weeks old) were acclimated to the new environment for a week (*n* = 6). Then the dorsal skin of mice (3 cm by 4 cm) was shaved and recovered for three days; mice exhibiting hair regrowth were excluded. From days 0–5, 62.5 mg of either Vaseline cream (blank group) or 5% imiquimod cream (other groups) were applied, after taking the pictures of back skin, to the shaved region once a day. For treatment, mice received 10^10^ CFU EcN or AIDEN-IL17 by gavage on days 0 and 3, and 2% Ara was provided in the drinking water throughout the entire treatment period. Anti-IL-17A mAbs was administered by subcutaneous injection on day 0 (10 mg·kg^−1^, 200 μL per mouse). To evaluate the treatment outcomes, the body weight was recorded daily. The PASI score was calculated as the sum of severity scores (0 = no symptoms), (1 = mild, 2 = moderate, 3 = severe and 4 = very severe) for erythema, desquamation and induration. On day 6, mice were sacrificed to obtain blood, skin tissues, and spleens. H&E staining, immunohistochemical staining (IL-17A^+^, PCNA^+^), immunofluorescence staining (Ki-67^+^, CD45^+^, CD11c^+^, F4/80^+^, CD11b^+^) of skin samples from all groups was performed. The spleens were weighted for calculating the spleen index (spleen weight/body weight). The blood sample and a portion of skin tissues that were not fixed from all groups were detected using ELISA kit to measure IL-1β, TNF-α, IL-17A, and IL-23 levels.

#### Flow cytometry assays of T lymphocytes isolated from mouse spleens

The spleens of mice from all four groups were collected to further evaluate the systematic immune response. Ground via a 200-mesh screen and lysed by RGB Lysis Buffer, the cell suspension was fluorescently labeled after incubation in dark for 30 min at 4°C as follows. The proportion of Tregs was detected using PE/Cyanine7-*anti*-mouse CD4, PE-*anti*-mouse CD45, and Alexa Fluor 488 anti-mouse FOXP3. The proportion of CD4^+^ T cells was detected using PE-*anti*-mouse CD45, FITC-*anti*-mouse CD3, and PE/Cyanine7-*anti*-mouse CD4. The proportion of CD8^+^ T cells was detected using PE-*anti*-mouse CD45, FITC-*anti*-mouse CD3, and APC-*anti*-mouse CD8.

#### Therapeutic efficacy of AIDEN-IL17 in DSS-induced mouse model

To establish DSS-induced IBD mice model, female C57BL/6 mice (7–8 weeks old) were acclimated to the new environment for a week (*n* = 5). On day 0, the mice were given 3% DSS and 2% Ara in drinking water for consecutive seven days. For treatment, different groups of mice were orally administered with PBS (blank and model groups), EcN (10^10^ CFU per mouse), AIDEN-IL17 (10^10^ CFU per mouse), and aIL17A@GEL (5 μg, 200 μL per mouse) on days 0 and 3. During the treatment period, body weight and DAI scores (calculated by weight loss score, stool consistency score and bleeding score, each from 0 to 4) were monitored daily. On day 7, mice were euthanized, and blood and colon tissues were harvested. H&E staining, immunohistochemical staining (MPO^+^, PCNA^+^, IL-17A^+^) of colon tissue samples from all groups was performed. The blood sample and a portion of colon tissues that were not fixed were detected using ELISA kit to measure TNF-α and IL-17A levels.

### Quantification and statistical analysis

Statistical analysis was performed using Prism 8 (GraphPad) software. Experimental data were expressed as means ± SEM. A two-tailed Student’s *t* test was used to determine statistical differences between two groups, whereas a multiple-group comparison was applied by ANOVA with Tukey’s multiple comparisons test. *p* < 0.05 was considered significance and represented as ∗. Other *p* values were represented as ∗∗*p* < 0.01, ∗∗∗*p* < 0.001, and ∗∗∗∗*p* < 0.0001.

## Supplemental information

Document S1. Figures S1–S10 and Table S1

Document S2. Article plus supplemental information
