# Supplementary material for: Engineered probiotics platform for oral delivery of antibody as a high-compliance alternative for immune-mediated inflammatory diseases
Source: Cell Rep Med. 2025 Dec 19;7(1):102523. doi: 10.1016/j.xcrm.2025.102523 (PMC12866112; doi:10.1016/j.xcrm.2025.102523)
Supplement: Document S1. Figures S1–S10 and Table S1 [file mmc1.pdf]

Cell Reports Medicine, Volume *7*

Supplemental information

Engineered probiotics platform for oral delivery of antibody as a high-compliance alternative for immune-mediated inflammatory diseases

Jia Liu, Lexuan Wang, Bingyu Pang, Yichenxi Shi, Yang Chen, Ruili Zhang, Shuai Shao, Chaoqiang Qiao, and Zhongliang Wang

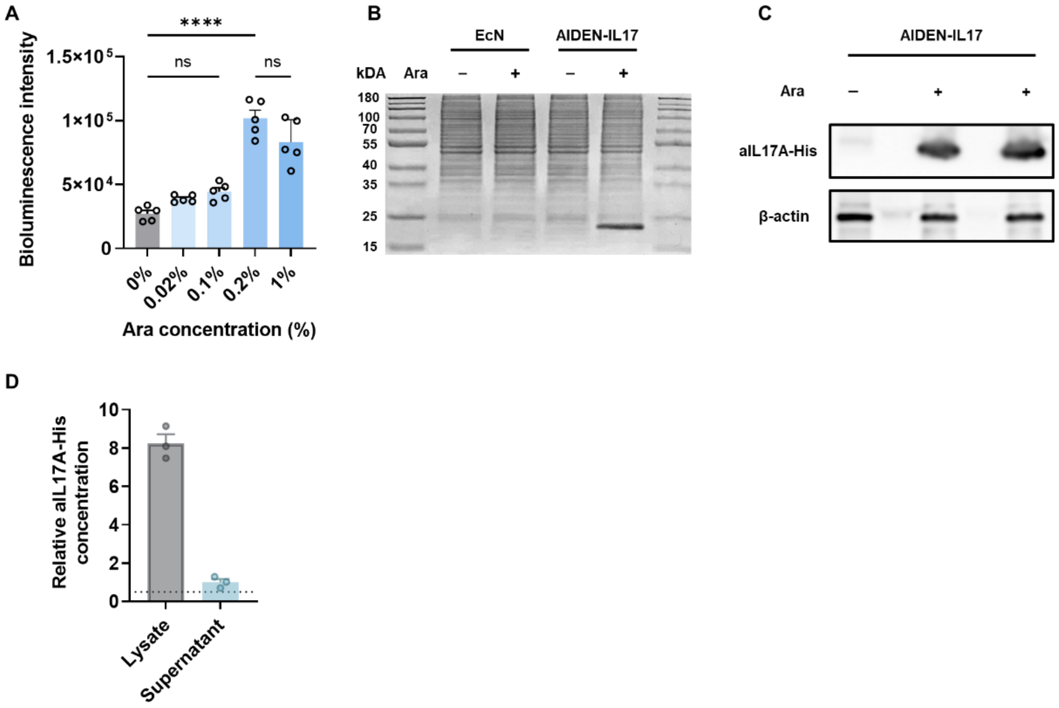

Figure S1. ***In vitro*** **characterization of AIDEN-IL17 and aIL17A. Related to Figures 1.** (A) Quantification of bioluminescence images of AIDEN-IL17-luc under different arabinose (Ara) conditions in Figure 1B (n = 5). (B–C) SDS-PAGE gel and Western blot images of EcN or AIDEN-IL17 lysate with or without Ara induction. (D) The secretion analysis of aIL17A-His by His-tag ELISA (n = 3). The dashed line represented detection limit. Data represent mean ± SEM. The *p* values in A were determined by one-way ANOVA with Tukey's multiple comparisons. ns, not significant, * $p < 0.05$, ** $p < 0.01$, *** $p < 0.001$, **** $p < 0.0001$.

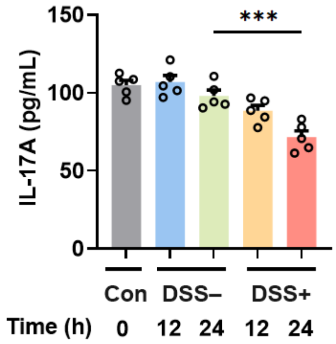

Figure S2. Intestinal permeability assessment at the cellular level. Related to Figure 1I. Remaining IL-17A concentration in the lower chamber after different incubation time of DMEM (Con) or supernatant from AIDEN-IL17 in the apical chamber. Data represent mean ± SEM (n = 5). The *p* values were determined by one-way ANOVA with Tukey's multiple comparisons. ns, not significant, * $p < 0.05$, ** $p < 0.01$, *** $p < 0.001$, **** $p < 0.0001$.

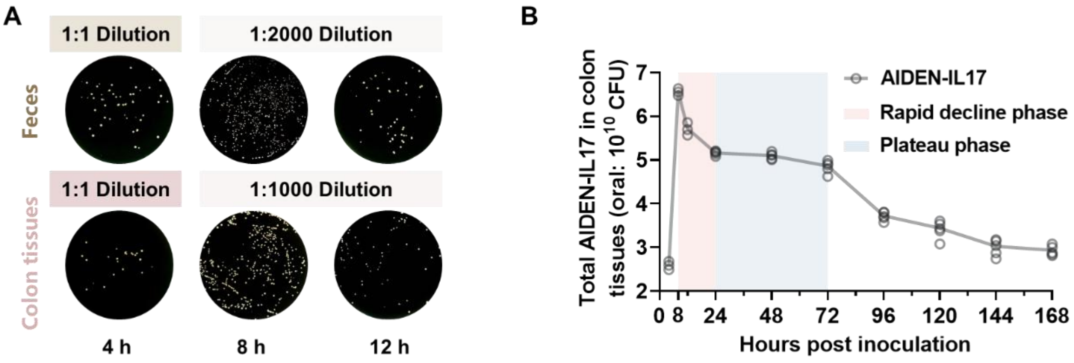

Figure S3. The gastrointestinal delivery kinetics of AIDEN-IL17. Related to Figures 2A and B. (A) Representative images of plate colony counting of colon tissues and feces samples, harvested from mice at 4, 8 and 12 hours (n = 3) (B) Quantification of plate colony counting of total colon tissues, harvested from mice at 4, 8 and 12 hours (n = 3) and 1–7 days (n = 5) after oral gavage with 10^{10} CFU AIDEN-IL17.

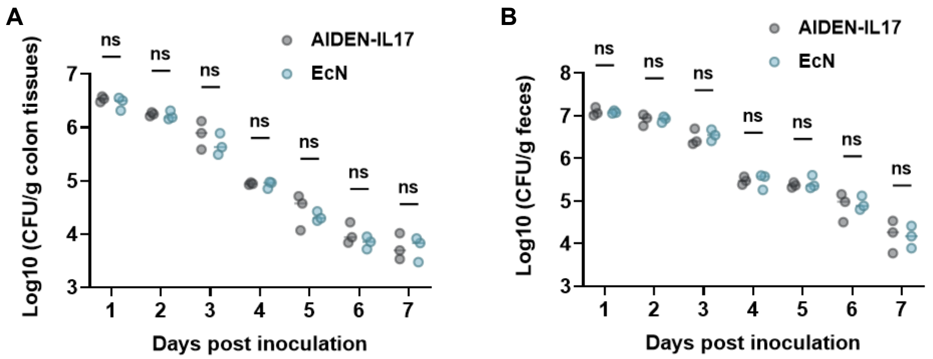

Figure S4. ***In vivo* persistence of AIDEN-IL17 in comparison with EcN. Related to Figure 2A.** Quantification of plate colony counting of colon tissues (A) and fecal samples (B), harvested from mice daily after oral gavage with AIDEN-IL17 or EcN. Data represent mean ± SEM (n = 3). The *p* values were determined by two-way ANOVA with Tukey's multiple comparisons. ns, not significant, * $p < 0.05$, ** $p < 0.01$, *** $p < 0.001$, **** $p < 0.0001$.

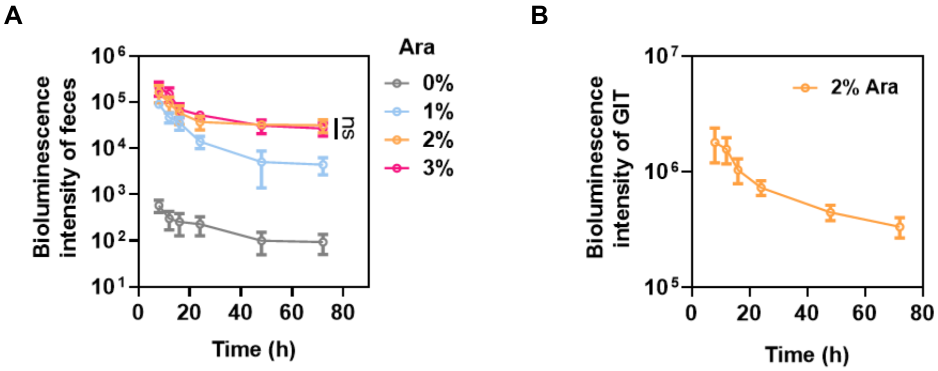

Figure S5. **Quantification of bioluminescence images of feces and colon tissues shown in Figures 2D and E.** Quantification of bioluminescence images of feces (A) and colon tissues (B) acquired at 8, 12, 16, 24, 48, and 72-hours post gavage. Data represent mean ± SEM (n = 5). The *p* values were determined by two-way ANOVA with Tukey's multiple comparisons. ns, not significant, * $p < 0.05$, ** $p < 0.01$, *** $p < 0.001$, **** $p < 0.0001$.

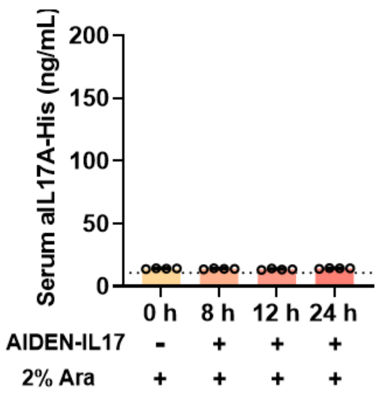

Figure S6. Serum levels of aIL17A-His. Related to Figure 2. Levels of aIL17A-His in serum of mice treated with AIDEN-IL17 and 2% Ara by His-tag ELISA (n = 4). Dashed lines indicate the limit of detection.

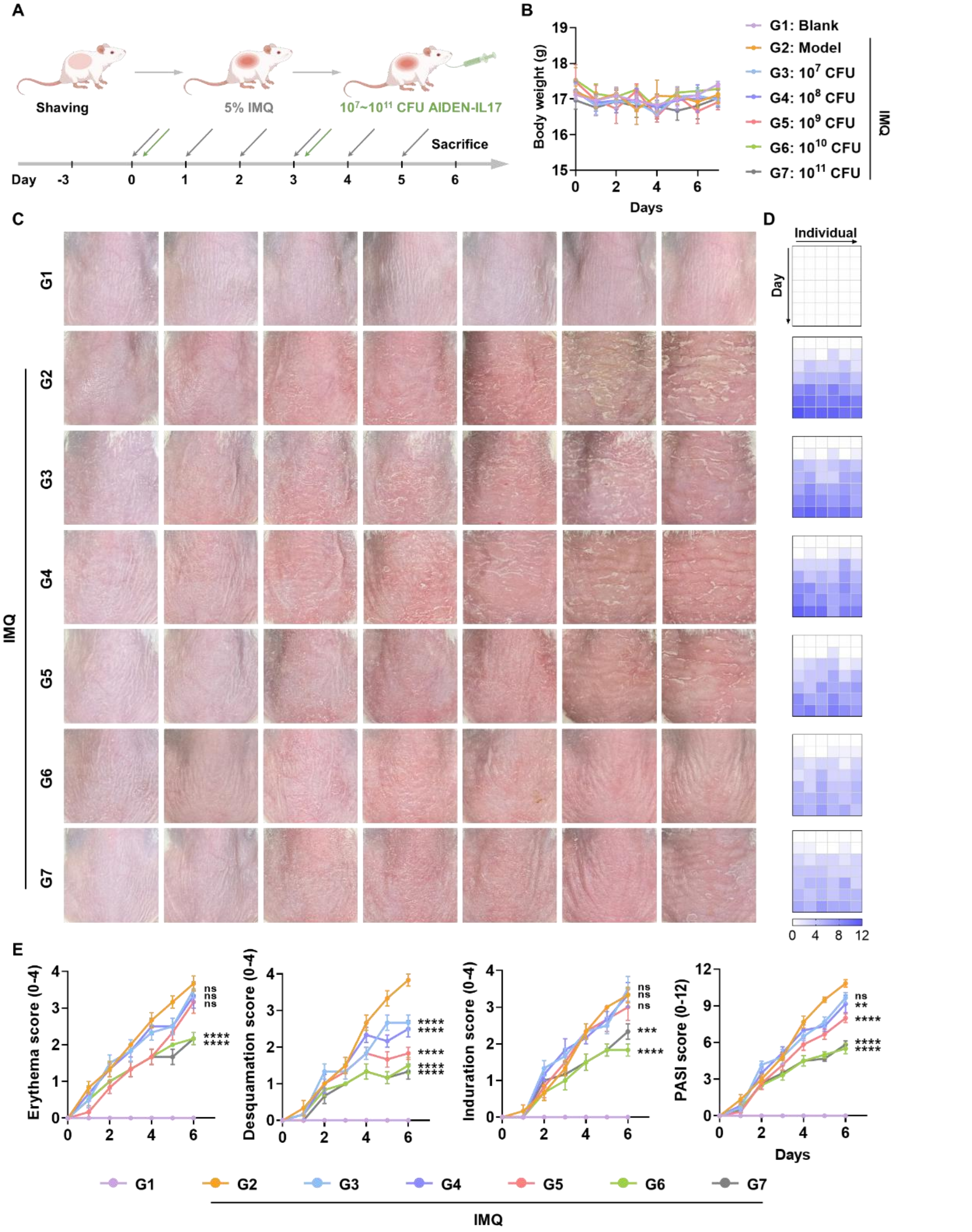

Figure S7. Dosage exploration of AIDEN-IL17 in psoriasiform dermatitis mice. Related to Figure 3. (A) Schematic representation of the experimental timeline. (B) Body weight changes. (C) Representative images of dorsal skins from days 0 to 6. (D) Heatmap of the total PASI scores of each mouse from days 0 to 6. (E) Erythema, desquamation, induration and total PASI scores each day. Comparisons were made between model group and AIDEN-IL17-treated group. Data represent mean ± SEM (n = 6). The *p* values were determined by two-way ANOVA with Tukey's multiple comparisons. ns, not significant, * $p < 0.05$, ** $p < 0.01$, *** $p < 0.001$, **** $p < 0.0001$.

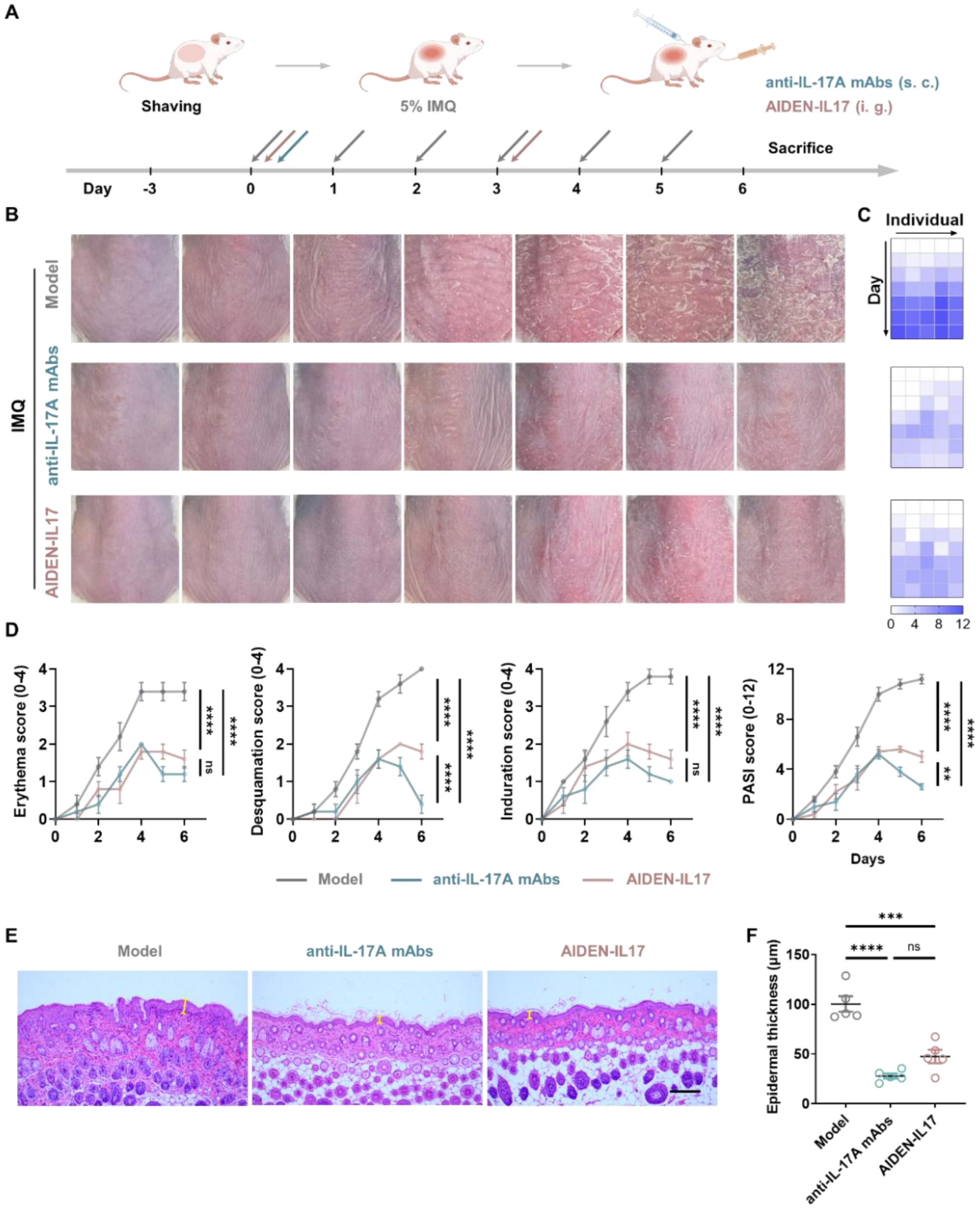

Figure S8. Efficacy of AIDEN-IL17 versus clinical psoriasis treatment. Related to Figure 3. (A) Schematic representation of the experimental timeline. (B) Representative images of dorsal skins of mice from days 0 to 6. (C) Heatmap of the total PASI scores of each mouse from days 0 to 6. (D) Erythema, desquamation, induration and total PASI scores each day. (E) Representative images of H&E staining of psoriasis skin. Scale bar, 200 μm. Yellow labeling highlighted the epidermis thickness. (F) Epidermal thickness quantification. Data represent mean ± SEM (n = 5). The *p* values in D were determined by two-way ANOVA with Tukey's multiple comparisons. The *p* values in F were determined by one-way ANOVA with Tukey's multiple comparisons. ns, not significant, * $p < 0.05$, ** $p < 0.01$, *** $p < 0.001$, **** $p < 0.0001$.

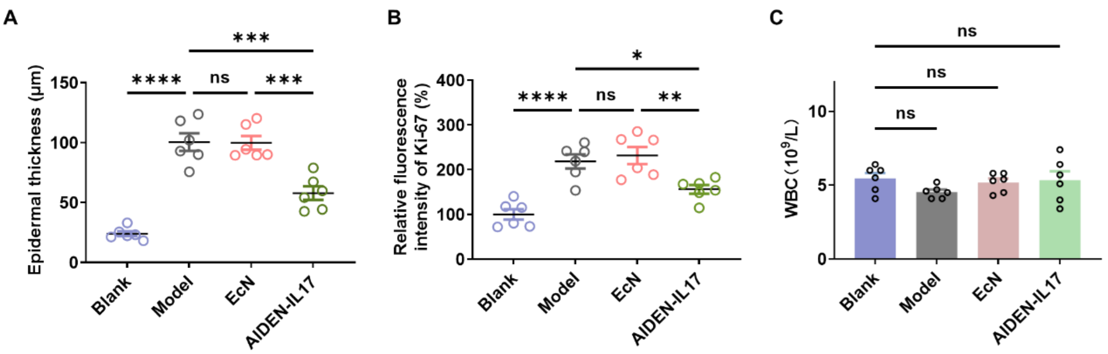

Figure S9. Quantification data of epidemic thickness, Ki-67 immunofluorescence and total WBC counting. Related to Figure 3. (A–B) Quantification data of epidemic thickness (A) and Ki-67 immunofluorescence (B) of psoriatic skin of mice in Figures 3E and F. (C) Total white blood cells counting in all groups, related to Figure 3J. Data represent mean ± SEM (n = 6). The *p* values were determined by one-way ANOVA with Tukey's multiple comparisons. ns, not significant, * $p < 0.05$, ** $p < 0.01$, *** $p < 0.001$, **** $p < 0.0001$.

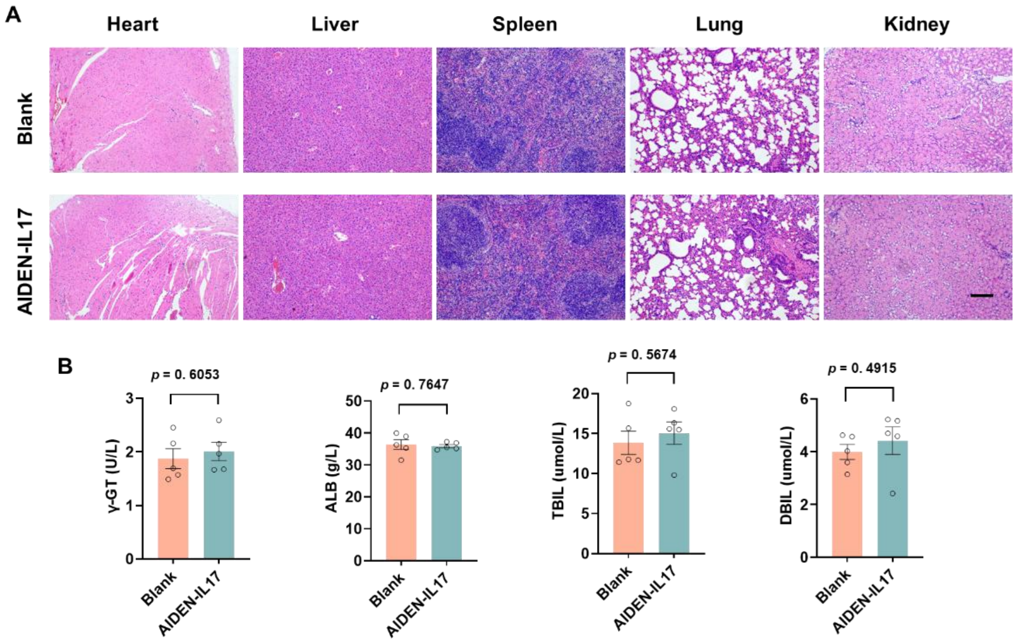

Figure S10. Safety characterizations in acute toxicity studies. Related to Figure 6. (A) Representative H&E-stained sections of main organs collected on day 14 in acute toxicity studies. Scale bar, 100 μm. (B) Serum levels of γ-glutamyl transpeptidase (γ-GT), albumin (ALB), total bilirubin (TBIL), and direct bilirubin (DBIL) in acute toxicity studies. The *p* values were determined by two-tailed Student's t-test. Data represent mean ± SEM (n = 5). ns, not significant, * $p < 0.05$, ** $p < 0.01$, *** $p < 0.001$, **** $p < 0.0001$.

Table S1. The amino acid sequence of aIL17A.

**PelB-aIL17A (VL-linker-VH)**
MKYLLPTAAAGLLLLAAQPAMAEIVMTQSPSSLSASVGDTVTITCRASHDINTYLAWFQQKPGKAPKSLIYAASNLQSGVPSKFSGSRSGTDFTLTISSLQPEDFATYYCLQYDTFPLTFGGGTKLEIKGGGGSGGGGSGGGGSEVQLLESGGGLVQPGGSLRLSCAASGFTFSPYSMMWVRQAPGKGLEWVSYIYSSGGWTTYADSVKGRFTISRDNSKNTLYLQMNSLRAEDTAMYYCARDTSLWFGDNDAFDIWGQGTMVTVSS
